# "The Play's the Thing" at Bath

**Published:** 1913-01-04

**Authors:** 


### "THE PLAY'S THE THING" AT BATH.

The Christmas festivities at the Royal Mineral Water Hospital gave satisfaction and enjoyment both to the patients and to the staff. In the women's day rooms the decorations were Japanese in character, the miniature lake in the centre being extremely realistic and tasteful. In that of the men airships, made by the staff and patients, were the centre of interest, the decorations being simple and showing good taste.

On Christmas Day dinner was carved by the past and present resident medical officers. The feast was admirably cooked and served. The patients cheered with right good will for the chaplain, doctors, matron, sisters, nurses, ward master, and last, but by no means less heartily, for the cook and her assistants as they entered bearing the Christmas pudding. The men were allowed whisky with their dinner, and wine was served to the women. The remainder of Christmas was devoted to services in the chapel and a smoking concert in the evening.

On Boxing Day all the patients were assembled in the men's day room to receive presents from the Christmas tree, which were distributed by the Mayor and Mayoress of Bath, Councillor and Mrs. Cooke. There were also present Mr. W. Handyside, the president of the hospital, and several members of the committee. The distribution was the scene of great enthusiasm; every patient received a present. A dance followed, in which most of the staff and patients joined, and soon a scene of frequent suffering was converted into a maze of dancing couples, not quite, however, "the dance's dangerous wreath" of Keats' sonnet. A most enjoyable evening ended at 9.30 by the singing of the National Anthem and "Auld lang syne."

On Friday evening, December 27, the sisters and nurses of the hospital presented a comedy entitled "A Mere Man." The piece was really a great success, especially as the ladies had no previous stage rehearsal; and, though all acquitted themselves well, special mention must be made of Sister Carter, who played the leading part and was repeatedly recalled at the conclusion of the performance. Songs were then sung by ladies and gentlemen who take an active interest in the hospital, and, the women having retired, the men patients finished the evening with a smoking concert. Christmas at our hospital has been a time of true enjoyment to patients and staff alike, and the latter, headed by our kindly and energetic Matron, have been untiring in their efforts to make the season long to be remembered by us all.

